# Computational Models of Anterior Cingulate Cortex: At the Crossroads between Prediction and Effort

**DOI:** 10.3389/fnins.2017.00316

**Published:** 2017-06-06

**Authors:** Eliana Vassena, Clay B. Holroyd, William H. Alexander

**Affiliations:** ^1^Donders Center for Cognitive Neuroimaging, Donders Institute for Brain, Cognition and Behaviour, Radboud University NijmegenNijmegen, Netherlands; ^2^Department of Experimental Psychology, Ghent UniversityGhent, Belgium; ^3^Department of Psychology, University of VictoriaVictoria, BC, Canada

**Keywords:** anterior cingulate cortex (ACC), effort, prediction error, computational models of ACC, computational modeling, effortful control

## Abstract

In the last two decades the anterior cingulate cortex (ACC) has become one of the most investigated areas of the brain. Extensive neuroimaging evidence suggests countless functions for this region, ranging from conflict and error coding, to social cognition, pain and effortful control. In response to this burgeoning amount of data, a proliferation of computational models has tried to characterize the neurocognitive architecture of ACC. Early seminal models provided a computational explanation for a relatively circumscribed set of empirical findings, mainly accounting for EEG and fMRI evidence. More recent models have focused on ACC's contribution to effortful control. In parallel to these developments, several proposals attempted to explain within a single computational framework a wider variety of empirical findings that span different cognitive processes and experimental modalities. Here we critically evaluate these modeling attempts, highlighting the continued need to reconcile the array of disparate ACC observations within a coherent, unifying framework.

## Introduction

Humans and other animals continually adapt their behavior in response to a rapidly changing environment, which requires speed and flexibility in evaluating environmental feedback. Research over the past two decades has identified anterior cingulate cortex (ACC) as a major neural hub for these computations (Rushworth et al., 2011), but the empirical evidence spans a wide variety of cognitive, affective and social functions (Bush et al., 2000; Nee et al., 2011; Gasquoine, 2013).

ACC is implicated in a lengthy list of processes (Shackman et al., 2011), including error detection (Gehring et al., 1993), conflict monitoring (Barch et al., 2001; van Veen and Carter, 2002), response selection (Holroyd and Coles, 2002), error likelihood (Brown and Braver, 2005), attention and task preparation (Luks et al., 2002; Aarts et al., 2008; Aarts and Roelofs, 2010), integration of outcome uncertainty and action values (Khamassi et al., 2015), reward prediction and prediction errors (Jessup et al., 2010; Silvetti et al., 2013; Vassena et al., 2014a), reward prediction errors experienced by others (Apps and Ramnani, 2014; Apps et al., 2016), prediction of effort required by the task (Vassena et al., 2014b; Chong et al., 2017), and perception of pain (Vogt, 2005; Fuchs et al., 2014). An automated machine-learning technique applied to a large data base also found ACC involvement in most tasks (Yarkoni et al., 2011). The diversity of contexts in which ACC activity has been observed has led to the ironic conclusion that ACC is involved in everything (Ebitz and Hayden, 2016), and that the search for a “unimodal” characterization may be unobtainable (Bush, 2009), a position that favors the possibility of a multitude of separate signals mapped to different areas in ACC (Bush et al., 2000; Kolling et al., 2016a,b). This ubiquitous ACC activation has stimulated a search for an overarching theoretical framework that can account for all of the data while complying with principles of parsimony, falsifiability, and neurobiological plausibility (Alexander and Brown, 2010b).

This paper reviews the history of computational models of ACC function, highlights the current state-of-the art, and discusses future directions. First, we summarize early seminal models that addressed single phenomena or relatively circumscribed sets of findings. These attempts mainly aimed at explaining fMRI and EEG data related to conflict and prediction errors. Second, we describe more recent models that account for growing evidence that ACC is implicated in effortful control (Walton et al., 2003, 2007; Vassena et al., 2014b; Holroyd and Umemoto, 2016; Klein-Flügge et al., 2016). Third, we describe models that broaden the explanatory scope to include lesion data and single-cell recordings under a shared underlying computational principle, none of which are comprehensive (see Table 1 for a schematic comparison across all models). Finally, we discuss current and future attempts to bridge the remaining gaps.

**Table 1:** Schematic comparison of all discussed models.

**Model**	**Publication**	**Model type**	**Effects**	**Data type**	**Species**
**EARLY MODELS**					
Conflict monitoring	Botvinick et al., 2001; Yeung et al., 2004	Connectionist	Conflict, errors	fMRI, EEG	Humans
Error likelihood	Brown and Braver, 2005	Rate-coded neurons	Conflict, errors	fMRI	Humans
Motor control filter	Holroyd and Coles, 2002, 2008	Reinforcement learning	Errors, prediction, reward prediction error	EEG	Humans
Volatility	Behrens et al., 2007	Bayesian	Volatility	fMRI	Humans
**RECENT EFFORT MODELS**					
Choice difficulty	Botvinick, 2007; Shenhav et al., 2014	Connectionist	Choice difficulty in decision-making	fMRI	Humans
Adaptive effort allocation	Verguts et al., 2015	Reinforcement learning	Physical and cognitive effort and cost-benefit trade off	fMRI, single-cell, lesion	Humans, rodents, monkeys
Expected value of control	Shenhav et al., 2013	Conceptual	Cognitive control and cost-benefit trade off in decision-making	fMRI	Humans
Synchronization by oscillations	Verguts, 2017a	Rate-coded neurons	Cognitive control driven by theta oscillations	(Intra-cranial) EEG	Humans
**RECENT UNIFYING MODELS**					
PRO	Alexander and Brown, 2011	Rate-coded neurons; reinforcement learning	Prediction and prediction error, conflict, error, pain	fMRI, EEG, single-cell	Humans, rodents, monkeys
PRO-Effort	Vassena et al., in press	= PRO	= PRO + effort	= PRO	Humans
PRO-Control	Brown and Alexander, 2017	= PRO	= PRO + foraging, choice difficulty	= PRO + lesion	Humans, monkeys
RVPM	Silvetti et al., 2011	Rate-coded neurons; reinforcement learning	Reward prediction and prediction error, conflict, error, volatility	fMRI, EEG, single-cell, lesion	Humans, monkeys
HRL-ACC	Holroyd and McClure, 2015	Reinforcement learning	Effort, task switching, hierarchical behaviors	Lesion	Rodents
RNN-ACC	Shahnazian and Holroyd, 2017	Connectionist	Distributed coding of extended action sequences, conflict, prediction errors	Singe-cell, fMRI, EEG	Rodents, humans
HER	Alexander and Brown, 2015	Predictive coding	as PRO, + dlPFC	fMRI, EEG, lesion, single-cell	Humans, monkeys
ACC-LPFC	Khamassi et al., 2011	Rate-coded neurons; reinforcement learning	Reward prediction error, salience, exploration-exploitation trade-off	Single cell, fMRI	Humans, monkeys

*For every model (early models, recent models effort models, and recent unifying models), the table provides first publication reference, model type (implementation), data type (data that the model was conceived to explain), and species to which this data belongs. Within model type, connectionist refers to the Parallel Distributed Processing approach (McClelland et al., 1987); reinforcement-learning refers to the approach described in Sutton and Barto (1998); rate-coded neurons refers to the approach described in Dayan and Abbot (2001)*.

## Early seminal models

The first computational accounts of ACC function underscored its involvement in different task settings and cognitive processes. Perhaps the most influential of these is the *conflict-monitoring model* (Botvinick et al., 2001), which identified ACC as a conflict monitor that increases in activation as a function of conflict between available response options. On this account, stimuli that are incompatible on two (or more) stimulus dimensions (such as word meaning and ink color in the Stroop task) can activate competing response channels (e.g., left and right button presses); conflict is defined as the multiple of the activity of these channels, signaling a need for increased top-down control. Although conflict-related activity has reliably been measured in ACC with fMRI and EEG (Botvinick et al., 1999; Yeung et al., 2004; Carter and van Veen, 2007; Roberts and Hall, 2008), findings in patients and non-human animal literature are controversial (Yeung, 2013). In particular, ACC lesions do not consistently impair the cognitive control adjustments that, according to the theory, should follow conflict detection (Swick and Jovanovic, 2002; Fellows and Farah, 2005; di Pellegrino et al., 2007; Sheth et al., 2012), and scant neurophysiological evidence from monkey single-cell recordings is highly debated (Nakamura et al., 2005; Cole et al., 2009; Ebitz and Platt, 2015). Subsequently, several groups reported neurophysiological and neuroimaging findings inconsistent with the conflict monitoring proposal (Amiez et al., 2006; Burle et al., 2008; Woodward et al., 2008; Hyafil et al., 2009; Kouneiher et al., 2009).

Brown and Braver (2005) later proposed the *error likelihood model*. According to this account, ACC associates errors with the stimulus context in which they occur, providing a means to predict the context-dependent likelihood of error commission. This model provided an early overarching attempt that explained both error and conflict activity. Subsequent experiments verified critical aspects of the model: as predicted, stimulus features associated with higher likelihood of errors, but not with conflict, elicited greater ACC activity (Brown, 2009). Failures to replicate the error likelihood effect (Nieuwenhuis et al., 2007) have been attributed to individual differences in risk-aversion (with more risk-averse subjects showing larger error-likelihood effects, Brown and Braver, 2007, 2008). One important limitation of this proposal was the inability to simulate the effect of unexpected errors (errors committed in contexts with low error likelihood elicit greater activity than errors in high error likelihood contexts, Brown and Braver, 2005).

An account by Holroyd and Coles (2002, 2008) proposed that ACC acts as a “motor control filter” that decides which action policy should be selected for a particular task. On this view, the value of action policies are learned via reward prediction error signals carried to ACC by midbrain dopamine system. These signals are proposed to encode discrepancies between expected and actual rewards that underlie the production of the error-related negativity (ERN) component of the event-related potential (ERP). Notably, this reinforcement learning model of the ERN (*RL-ERN theory*) shifts the role of ACC from the evaluative domain (i.e., detecting response conflict or error likelihood) to the action selection domain, explaining how ACC signals affect behavior. Aspects of the RL-ERN theory have received strong empirical support (Walsh and Anderson, 2012; Sambrook and Goslin, 2015; Holroyd and Umemoto, 2016) and are compatible with evidence of ACC encoding action values in uncertain environments (e.g., during foraging, Kolling et al., 2012, 2016b). However, the explanatory scope of this proposal was mainly limited to EEG data, and is not easily translated to fMRI (cf. Holroyd et al., 2004; Nieuwenhuis et al., 2005; Becker et al., 2014; Ferdinand and Opitz, 2014). Furthermore, this model proposed a general role for ACC as a motor control filter, describing a high-level hierarchical mechanism for action selection, but did not make specific predictions about how reward and error signals regulate behavior.

Finally, Behrens et al. (2007) proposed that ACC is sensitive to the *volatility* of environmental outcomes. This proposal holds ACC responsible for detecting how rapidly reward contingencies change over time. The model provides a mechanism by which organisms can flexibly adapt their learning rate (i.e., the speed at which current knowledge of the world is updated with new information). The volatility measure computed by ACC is used to adjust this learning rate in order to optimize subsequent decision-making. Furthermore, according to the authors the volatility signal is dissociable from prediction errors signals, thus implicitly postulating co-existence of difference signals within ACC. One limitation of this proposal is that while the volatility signal is proposed to influence learning rate at the time of feedback, this model does not address how ACC contributes to action selection.

Although these computational models provided the first steps toward a mechanistic understanding of ACC function, they share a limitation in having been mainly conceived to explain one type of experimental data. This aspect is perhaps particularly problematic when based on fMRI data: BOLD measurements provide an indirect and possibly biased means for assessing neuronal activity (Logothetis, 2002, 2008), and further, increases in activity in ACC may reflect synaptic activity from projecting regions rather than firing by local neurons in ACC.

## Recent models related to effort and difficulty

Recent findings have drawn attention to the central role of ACC in control processes requiring effort. Generally, ACC seems to be more active when subjects prepare for difficult or effortful tasks, even in absence of error, conflict, and choice (Mulert et al., 2005; Aarts et al., 2008; Vassena et al., 2014b). ACC lesions impair decisions that evaluate trade-offs between effort expenditure and reward value in non-human animals (Walton et al., 2002, 2003, 2007), and are associated with motivational impairments and apathy in humans (e.g., Devinsky et al., 1995; Holroyd and Umemoto, 2016).

Botvinick (2007) anticipated this line of research with a simple model proposing that the conflict signal may drive effort avoidance, thus linking the conflict monitoring theory with decision-making. This idea was later extended to the proposal that ACC codes for *choice difficulty* (i.e., conflict between choice options), based on the observation that BOLD-fMRI ACC activity during decision-making negatively correlates with value differences between available options (Pochon et al., 2008; Shenhav et al., 2014). While not explicitly modeling effort, this proposal is one of the first to point to a role of ACC in coding difficulty.

The *adaptive effort allocation model* by Verguts et al. (2015) addresses the role of ACC in effortful control explicitly, accounting for the empirical finding that expectation of effort in absence of choice or conflict is associated with increased ACC activity (Vassena et al., 2014b). On this account, ACC units implement a “boosting” mechanism, biasing behavior toward more effortful options when it is worth it (i.e., when they are predicted to procure a large enough reward). The model predicts that boosting increases the signal-to-noise ratio in task-related brain areas, thereby ensuring successful task completion. Although carrying a cost that increases linearly as a function of task difficulty, the boosting mechanism ensures sufficient cognitive or physical effort is deployed to obtain the reward at stake. This model effectively implements an “effort module” that can influence other cortical regions as appropriate to the task at hand.

In line with the adaptive effort allocation model, the *expected value of control* framework (EVC) proposes that ACC computes the value of exerting cognitive control (Shenhav et al., 2013), integrating a variety of signals (including conflict, reward, costs, effort, choice difficulty, and so on) in order to determine the degree of control worth applying over task performance. The EVC theory hypothesizes a role for ACC in calculating the “value of control” based on a combination of multiple different signals, some of which have also been ascribed to ACC; the EVC framework thus postulates calculations of the expected value of control as an additional role of ACC rather than a single mechanism explaining the variety of signals observed within the region. This framework has recently contributed to a lively and ongoing debate on the neural mechanisms of *foraging*, with a series of experiments inspired by the ecology literature pointing to a critical role for ACC: according to this proposal, ACC activity reflects the relative value of foraging, i.e., of leaving a known environment in order to explore a new environmental patch, which is associated with higher uncertainty but also potentially higher rewards (Kolling et al., 2012, 2016b). Whereas Shenhav and colleagues suggested that such a foraging signal reflects choice difficulty in a foraging context (Shenhav et al., 2014), Kolling and colleagues proposed that choice difficulty and foraging are dissociable and coded in segregated sub-regions of ACC. Overall, this proposal would appear to be consistent with Shenav and colleague's EVC account, assuming an additional signal in ACC that codes for foraging and inputs to the calculation of the value of exerting control. While providing a theoretical framework with potentially wide explanatory scope, the EVC theory has yet to be translated to a detailed computational framework with testable (viz. falsifiable) predictions. Although developed by a different group of investigators, one could consider the Adaptive Effort Allocation model (Verguts et al., 2015) as a possible computational instantiation of the EVC framework.

Another recent model has specified a role for ACC in synchronizing neural oscillations across brain areas (Verguts, 2017a). This complementary perspective suggests that ACC exerts top-down control with bursts of activity in the theta frequency band that synchronize task-related areas throughout cortex, resulting in more efficient cortical communication. This proposal aligns with evidence that theta oscillations originating in ACC reflect effortful control (Holroyd, 2016; Holroyd and Umemoto, 2016), and is unique in describing the role of ACC in cognitive control in terms of synchronizing neural oscillations.

Overall, these recent proposals complement their predecessors in so far as they account for effort and control effects, which were only partially explained by previous models. However, the explanatory scope remains limited to the domain of effort-based behavior, and mostly neglects to account for data across different experimental models within a single framework.

## Recent unifying trends

In parallel with the development of ACC models of effort processing, several computational models have tried to account for a wider array of empirical findings within a single, overarching theoretical framework. In particular, whereas many previous theories focused on explaining functional neuroimaging data, recent models have widened their scope to include lesion and neurophysiological data. An early step in this direction was the *Predicted Response Outcome* (PRO) model (Alexander and Brown, 2011), which assigns to ACC the role of a stimulus-action-outcome predictor. In this account, ACC predicts the likelihood of upcoming actions and outcomes based on stimulus input from the environment; the predictions are then compared with actually experienced outcomes to produce a prediction error when an expected outcome and an actual outcome do not match. This error signal informs the prediction units, updating the predictions for future reference. Therefore, ACC is mainly sensitive to the (un)predictability of outcomes, regardless of their affective valence, as well as the unpredicted non-occurrence of these outcomes. Under this simple computational principle, the PRO model is able to simulate a wide variety of empirical findings, including sensitivity of ACC to conflict, errors, reward prediction errors and pain, for both neuroimaging and single-cell data (Alexander and Brown, 2014; Jahn et al., 2014, 2016). Simply put, the proposed mechanism for monitoring the (un)predictability of outcomes and their deviations from expectation provides a unifying framework for understanding such a diverse array of empirical findings.

A similar framework, the *Reward Value and Prediction Model* (RVPM), independently proposed by Silvetti et al. (2011), implements a comparable prediction mechanism in ACC, with one major difference: according to the RVPM, ACC predicts the value of future outcomes, but only when reward is at stake. By contrast, according to the PRO model, ACC predicts the likelihood of any outcomes (even for events that have no intrinsic value; Alexander and Brown, 2014). Like the PRO model, the RVPM successfully explains a wide range of data based on the principle of prediction and prediction errors (Silvetti et al., 2013). However, while both models have the potential to be extended to the domain of effortful control, a translation to the effort domain has been recently proposed only for the PRO model (Vassena et al., in press).

While neuroimaging studies have suggested a critical role for ACC in performance monitoring and control, neuropsychological reports on patients with ACC lesions do not show the dramatic impairments one would expect based on these findings (Yeung, 2013). Partly for this reason, Holroyd and Yeung (2012) proposed that ACC selects and maintains extended, goal-directed action sequences, rather than instigate moment-to-moment changes in behavior following conflicts and errors. On this view, ACC impairments would impact complex high-level goal-pursuit rather than individual, low-level actions. These ideas were implemented by Holroyd and McClure (2015) in a 3-level, hierarchical reinforcement learning (HRL) model of rodent behavior (*HRL-ACC model*). Here a mid-level module associated with a caudal region of ACC selects tasks for execution and, in line with the literature on ACC involvement in effortful control, applies a control signal that attenuates effort-related costs incurred by a low-level action selection mechanism, ensuring that the task is completed successfully. Likewise, a high-level module located in rostral ACC selects the “meta-task” for execution and applies a control signal over caudal ACC that attenuates effortful costs incurred in task switching, facilitating shifts between different task strategies. The control levels are regulated according to tonic dopamine levels in cortex, which are assumed to code for average reward rate. On this view, the role of ACC in foraging relates to increased control by caudal ACC for exploiting a current patch, vs. increased control by rostral ACC for switching to alternative patches. Crucially, the model accounts for the effects of ACC lesions on rodent behavior and is broadly compatible with comparable observations in humans with ACC lesions.

Although the HRL-ACC model implements some of the key aspects of the proposal that ACC is responsible for selecting and motivating the execution of extended behaviors (Holroyd and Yeung, 2012), it does not directly account for neuroimaging and single-cell evidence in ACC. To address this gap, Shahnazian and Holroyd (2017) simulated the role of ACC in the production of goal-directed action sequences using a recurrent neural network (RNN) approach that had been previously used to simulate the production of hierarchical action sequences (Botvinick and Plaut, 2004). This *RNN-ACC model* predicts each successive event in the sequence and, like the PRO (Alexander and Brown, 2011) and RVPM (Silvetti et al., 2011) models, generates prediction errors when the events are unexpected, as observed in functional neuroimaging and EEG data (Botvinick et al., 2001; Alexander and Brown, 2010a; Wessel et al., 2012). Further, unique to this model, the predictions are hypothesized to be encoded as highly distributed representations across ensembles of neurons in ACC, as observed in studies of non-human animals (e.g., Ma et al., 2014a,b). Nevertheless, although this model is inspired by the broader theoretical framework of the HRL-ACC theory (Holroyd and Yeung, 2012), it has yet to be integrated with the HRL-ACC model (Holroyd and McClure, 2015).

## Discussion

Modeling ACC function faces the challenge of accounting for a multitude of empirical findings within a single coherent framework. Although progress has been made over the last two decades in explaining a wider array of empirical findings associated with different experimental techniques (Verguts, 2017b), the debate about underlying computational principles remains lively. We see several outstanding conceptual issues that remain to be resolved, many of which lie at the crossroads between predictive mechanisms and effortful control.

A first issue is that effortful control has been addressed with dedicated mechanisms that are constrained in scope. For example, the *adaptive effort allocation model* (Verguts et al., 2015) does not account for ACC activity related to predictions and prediction errors. Although the proposed mechanism could be implemented with separate ACC units, with an RVPM or PRO-like module computing prediction error signals and a boosting module driving adaptive effort exertion, this potential integration remains speculative. Conversely, we have recently proposed a possible translation of the PRO model to the effort domain (Vassena et al., in press). This model explains effort-related effects in terms of outcome prediction and error signaling, where required effort is considered as an outcome of the choice to engage in the task. As such, the PRO model explains increased ACC activity following a choice to engage in an effortful task as deriving from the “surprise” of choosing a high-effort trial. The model reconciles effort-related neuroimaging data in ACC with the variety of findings already explained by the PRO model under the unifying principles of prediction and prediction error (Jahn et al., 2014, 2016).

The ACC-HRL theory is characterized by similar tensions between different sources of evidence (Holroyd and Yeung, 2012). On the one hand, the ACC-HRL model accounts for the effects of ACC damage on rodent behavior (Holroyd and McClure, 2015) and is compatible with both neuroimaging data related to effort and control, and electrophysiological evidence of ACC reward prediction errors (Holroyd and Umemoto, 2016; Umemoto et al., 2017). On the other hand, it does not explicitly address the conflict and surprise signals commonly observed in ACC, nor any ACC single-cell data in non-human animals. Conversely, the ACC-RNN model accounts for surprise and error signals while simultaneously describing single-cell activity as arising from distributed representations of ensembles of ACC cells while animals execute goal-directed action sequences (Shahnazian and Holroyd, 2017). However, this architecture does not fully exploit hierarchical representations, nor utilizes reward signals for regulating control levels. A natural next step would be to integrate these approaches in line with recent examples (e.g., Cooper et al., 2014), using a more biologically-realistic network that incorporates finer temporal dynamics into the unit activity (Sussillo, 2014).

A related issue concerns the neurobiological plausibility of the proposed accounts across species. Some proposals are based on (or at least compatible with) neurophysiological and lesion findings in non-human primates and/or rodents, while others are mainly based on human EEG or fMRI findings (see Table 1). A comprehensive account of ACC function should bridge apparent inconsistencies across experimental modalities, linking the indirect results of neuroimaging with single-neuron activity (as attempted for example by the PRO, RVPM, and ACC-RNN models).

Second, an important challenge in modeling ACC function is to account not only for the monitoring processes in which ACC is involved (such as conflict and error detection), but also how these computations modulate subsequent behavior. A recent proposal extended the PRO model in this direction (*PRO-Control*, Brown and Alexander, 2017), suggesting how prediction and error signals computed in ACC can serve as the basis for proactive and reactive control. While the PRO-Control assigns the same computations to ACC as in previous iterations of the model (Alexander and Brown, 2011, 2014; Alexander et al., 2015), it is able to account for additional behavioral and imaging effects related to deploying control, including effects of foraging value and choice difficulty (cf. the controversy mentioned above, Kolling et al., 2016b; Shenhav et al., 2016). Another modeling approach that may explain how ACC signals drive behavior is the meta-learning perspective. For example, Verguts and Notebaert (2008, 2009) proposed that control may emerge as a result of Hebbian learning, while Khamassi et al. (2013) proposed that ACC monitoring function determines learning rate, thus informing exploration-exploitation trade-offs in other brain regions.

A further challenge is to account not only for the function of ACC during normal behavior, but also in circumstances in which behavior and ACC function is impaired, as in the case of lesions to ACC (e.g., Devinsky et al., 1995; Fellows and Farah, 2005; Kennerley et al., 2006; Walton and Mars, 2007; Camille et al., 2011; Tsuchida and Fellows, 2013) or in clinical disorders such as, e.g., substance dependence, depression, or obsessive-compulsive disorder (Rive et al., 2013; Gowin et al., 2014; Barch et al., 2016). Initial work has attempted to link existing computational accounts of ACC to behavioral dysfunction (Alexander et al., 2015; Holroyd and Umemoto, 2016; Vassena et al., in press), and additional efforts in this direction may further refine understanding of the role of ACC during both normal and abnormal behavior.

Finally, a larger objective is to develop theories that integrate ACC function into the broader network of brain areas involved in control and decision-making. While the interaction of ACC with additional brain regions, including dorsolateral prefrontal cortex (dlPFC) and basal ganglia, was incorporated into early models of ACC (Botvinick et al., 2001; Holroyd and Coles, 2002; Kerns et al., 2004), the nature of this interaction has remained mostly an issue of secondary concern. Although the basal ganglia are recognized as a hub for interaction of motor, cognitive and motivational processes, modeling efforts to describe interactions between these areas and ACC are surprisingly scarce (but see, e.g., Hikosaka and Isoda, 2010; Cockburn and Frank, 2011). The ACC-HRL theory takes a step in remediating this deficit by proposing how ACC interacts with dorsolateral prefrontal cortex, orbitofrontal cortex, the striatum, and other brain areas (Holroyd and Yeung, 2012; Holroyd and McClure, 2015; Holroyd and Umemoto, 2016). Likewise, the *Hierarchical Error Representation model* (HER, Alexander and Brown, 2015) examines the interaction of ACC and dlPFC through a hierarchical predictive coding framework, which replicates the PRO model at hierarchical levels that map onto a putative rostrocaudal gradient of abstraction in prefrontal cortex (Badre and D'Esposito, 2007; Taren et al., 2011; Nee and D'Esposito, 2016), and that sub-serve different higher-order cognitive functions. The HER model explains the function of large regions of PFC as primarily concerning the computation, representation, and manipulation of quantities derived from prediction error. Khamassi et al. (2011) have also proposed a computational account that simulates cellular activity in both ACC and LPFC, predicting that feedback-related signals in ACC modulate exploration-exploitation trade-off in LPFC during decision-making. This proposal implements dopamine input to ACC not only during feedback-related reward prediction errors, but also at the occurrence of any salient event, reconciling previous proposals based on dopaminergic input (cf. Holroyd and Coles, 2002, 2008) with neurophysiological evidence that dopamine also responds to salient but non-rewarding events (Horvitz, 2000).

These modeling attempts highlight important objectives for future models of ACC. As a first goal, existing ACC models with relatively wide explanatory power should be extended to other domains, especially effortful control, that heretofore have been the target of more constrained models. As a second goal, models of ACC should be integrated into more comprehensive accounts that explain how the ACC interacts with other brain regions. The benefits of such an approach are two-fold. First, the wider scope of empirical data predicted by the models would provide means for their falsification. In much the same way that the failure to observe single neurons encoding conflict signals paved the way for new models that could account for single-unit activity as well as for the activity of neural ensembles, the possible failure of current models to account for effortful decision-making may point the direction toward even more comprehensive accounts. Second, in the event that existing models can be extended to account for motivational effects in ACC, they provide a basis for understanding interactions with additional brain regions, providing insights into the function of the brain beyond cingulate cortex alone.

## Author contributions

EV, CBH, and WHA reviewed the literature, drafted the manuscript and provided critical comments.

### Conflict of interest statement

The authors declare that the research was conducted in the absence of any commercial or financial relationships that could be construed as a potential conflict of interest.
